# Local Suppression of T Cell Responses by Arginase-Induced L-Arginine Depletion in Nonhealing Leishmaniasis

**DOI:** 10.1371/journal.pntd.0000480

**Published:** 2009-07-14

**Authors:** Manuel Modolell, Beak-San Choi, Robert O. Ryan, Maggie Hancock, Richard G. Titus, Tamrat Abebe, Asrat Hailu, Ingrid Müller, Matthew E. Rogers, Charles R. M. Bangham, Markus Munder, Pascale Kropf

**Affiliations:** 1 Department of Cellular Immunology, Max-Planck-Institute for Immunobiology, Freiburg, Germany; 2 Department of Immunology, Faculty of Medicine, Imperial College London, London, United Kingdom; 3 Lipid Biology in Health and Disease Research Group, Children's Hospital Oakland Research Institute, Oakland, California, United States of America; 4 Imperial College Healthcare NHS Trust, Chelsea and Westminster Hospital, London, United Kingdom; 5 Microbiology, Immunology, and Pathology Department, Colorado State University, Fort Collins, Colorado, United States of America; 6 Department of Microbiology, Parasitology and Immunology, University of Addis Ababa, Addis Ababa, Ethiopia; 7 Department of Hematology, Oncology, and Rheumatology, University Hospital Heidelberg, Heidelberg, Germany; Hebrew University, Israel

## Abstract

The balance between T helper (Th) 1 and Th2 cell responses is a major determinant of the outcome of experimental leishmaniasis, but polarized Th1 or Th2 responses are not sufficient to account for healing or nonhealing. Here we show that high arginase activity, a hallmark of nonhealing disease, is primarily expressed locally at the site of pathology. The high arginase activity causes local depletion of L-arginine, which impairs the capacity of T cells in the lesion to proliferate and to produce interferon-γ, while T cells in the local draining lymph nodes respond normally. Healing, induced by chemotherapy, resulted in control of arginase activity and reversal of local immunosuppression. Moreover, competitive inhibition of arginase as well as supplementation with L-arginine restored T cell effector functions and reduced pathology and parasite growth at the site of lesions. These results demonstrate that in nonhealing leishmaniasis, arginase-induced L-arginine depletion results in impaired T cell responses. Our results identify a novel mechanism in leishmaniasis that contributes to the failure to heal persistent lesions and suggest new approaches to therapy.

## Author Summary

*Leishmania* parasites are obligate intracellular pathogens that predominantly invade macrophages. Instruction of macrophages by T cell-derived signals is required to control parasite growth. Here we show that arginase, an enzyme induced in *Leishmania*-infected macrophages, is highly expressed at the site of pathology in nonhealing lesions and causes local depletion of L-arginine, an amino acid that is essential for efficient T cell responses. This local reduction in L-arginine impairs the capacity of T cells in the lesion to proliferate and to produce interferon-γ, one of the signals required for parasite killing. Cure of *Leishmania* infection by drug treatment is accompanied by a reduction in arginase activity and restoration of T cell effector functions. Furthermore, inhibition of arginase, as well as injection of L-arginine, reverses immunosuppression and results in more efficient control of parasite replication. Our results identify a novel mechanism accounting for ineffective T cell responses in nonhealing leishmaniasis.

## Introduction

The leishmaniases, a group of vector-borne parasitic diseases, represent a major public health problem worldwide. Currently, the diseases affect an estimated 12 million people in 88 countries, and approximately 350 million people are at risk. Leishmaniases present with a wide range of symptoms, ranging from the self healing cutaneous form, which produces skin ulcers; to the mucocutaneous form, which leads to the destruction of mucous membranes of the mouth, throat, nose and neighbouring tissue; to the visceral form, the most severe form of leishmaniasis, in which the mortality rate can be as high at 100%. The leishmaniases belong to the most neglected tropical diseases, affecting the poorest populations, for whom access to diagnosis and effective treatment are most difficult. Much effort has been put into the discovery of new drugs for the treatment of this pathology, but still the most widely used drugs remain the pentavalent antimonials, which were introduced 50 years ago. However, these drugs have many limitations, such as the long course of treatment, severe side effects and development of resistance. No efficient vaccine is available to date (http://www.who.int/leishmaniasis/en/).

In all forms of leishmaniasis, both immunity and pathology are predominantly mediated by T lymphocytes. Experimental studies in inbred strains of mice with *Leishmania (L.) major* have established the current paradigm of T helper (Th) cell subset involvement in infectious diseases. In this paradigm, control of infection and healing have been associated with a polarized Th1 response whereas non-healing has been ascribed to an interleukin (IL)-4-dominated polarized Th2 response [1],[2]. However, the regulation of immune responses against *Leishmania* parasites is complex and Th2 dominance does not fully explain nonhealing [3],[4]. Furthermore, in human leishmaniasis, the different clinical outcomes do not appear to be solely associated with Th1- or Th2-type responses. Indeed, whereas IL-4 has been shown to be higher in the plasma of patients with visceral leishmaniasis (VL) [5],[6], several proinflammatory cytokines such as IL-1, IL-6, IL-12, interferon(IFN)-γ and tumor necrosis factor(TNF)-α are also elevated [7],[8].

One of the main immunological features of the active form of visceral [9],[10],[11] and nonhealing cutaneous leishmaniasis (CL) [12] is a pronounced immunosuppression, as shown by the inability of peripheral blood mononuclear cells (PBMCs) to proliferate and produce IFN-γ in response to antigenic challenge. The mechanisms leading to this suppression are not fully identified and it is not clear whether there are a cause or a consequence of nonhealing disease.

*Leishmania* are obligate intracellular parasites in their mammalian host: they survive and replicate predominantly in macrophages. Depending on the balance of two inducible enzymes, nitric oxide synthase 2 (iNOS) and arginase, macrophages can either kill parasites or support their growth. These two enzymes use a common substrate, L-arginine, and are competitively regulated by type 1 and type 2 cytokines [13],[14]. The type 1 cytokine, IFN-γ, induces classical activation of macrophages and expression of iNOS that in turn oxidizes L-arginine in a two-step process into nitric oxide (NO) - a metabolite responsible for parasite killing. The key type 2 cytokine, IL-4, results in alternative activation of macrophages and the induction of arginase that hydrolyzes L-arginine to urea and ornithine. The latter is the main intracellular source for the synthesis of polyamines, which are necessary for parasite growth [15].

The metabolism of L-arginine by arginase is emerging as a crucial mechanism for the regulation of immune responses. Arginase 1 has been shown to impair T cell responses by reducing the bioavailability of L-arginine: high arginase activity expressed by myeloid cells results in increased uptake and hydrolysis of extracellular L-arginine into the cells, thereby causing a reduction of L-arginine levels in the microenvironment. In turn, this decrease in L-arginine results in T cell hyporesponsiveness [16],[17],[18],[19],[20],[21],[22],[23]. This T cell dysfunction is directly attributed to L-arginine starvation, arresting cells in the G0-G1 phase [19]. Arginase-mediated L-arginine deprivation has been shown to cause T cell hyporesponsiveness in a variety of pathological and physiological responses [22],[24],[25]. High arginase expression has been associated with a variety of diseases such as chronic inflammation [23], asthma [26], psoriasis [27] and infectious diseases [15],[28],[29],[30].

We and others have recently shown that *in vivo* uncontrolled replication of *Leishmania* parasites at the site of pathology in nonhealing BALB/c mice correlates with abnormally high levels of arginase activity [15],[31]. In the present study we tested the hypothesis that excessive arginase activity at the site of pathology contributes to persistent nonhealing leishmaniasis by causing local suppression of T cell responses.

## Methods

### Mice

6–8 week old female BALB/c and CBA mice (Charles River, UK) were kept in individually vented cages. Animal colonies, screened regularly for mouse pathogens, consistently tested negative. Animal experiments were performed in accordance with Home Office and institutional guidelines.

### Experimental infection with *L. major* parasites

For infections, 2×10^6^ stationary phase *L. major* LV39 (MRHO/SU/59/P-strain) promastigotes or 1×10^5^ metacyclic *L. major* LV39 (MRHO/SU/59/P-strain) isolated as described in [32] were injected subcutaneously (s.c.) into the footpad, and lesions monitored as described [33].

Nanometer-scale, apolipoprotein-stabilised phospholipid bilayer disk complexes (nanodisks; ND) harboring amphotericin B (AMB) [34],[35] were injected intraperitoneally (i.p.) in *L. major* infected BALB/c mice at a concentration of 5 mg/kg (as AMB) on days 1, 2, 7, 14, 21 and 28 post infection.

To inhibit arginase *in vivo*, mice were treated i.p. daily, starting on the day of infection and throughout the course of infection with 1 mg of *N* ^ω^-hydroxy-nor-L-arginine (nor-NOHA, Bachem, Bubendorf, Switzerland) in 0.1 ml of PBS.

L-arginine (L-arginine-monohydrochloride, Roth) was injected i.p. at a concentration of 10 mg/100 µl, three times a week, starting on day 15 post infection.

### Determination of parasite load

The number of living *L. major* parasites in infected tissues was determined using a parasite limiting dilution assay as described [33].

### Determination of arginase activity

Arginase activity was determined using 25 µl of tissue homogenate, as described [15]. Briefly, 25 µl of tissue homogenate was solubilized with 25 µl of a solution containing 0.1% Triton X-100/10 mM MnCl_2_/25 mM Tris-HCl and the enzyme was activated by heating for 10 min at 56°C. Arginine hydrolysis was achieved by incubating the lysate with 50 µl of 0.5 M L-arginine (pH 9.7) at 37°C for 15–120 min. The reaction was stopped with 400 µl of H_2_SO_4_ (96%)/H_3_PO_4_ (85%)/H_2_O (1/3/7, v/v/v). Urea concentration was measured at 550 nm after addition of 20 µl α-isonitrosopropiophenone (dissolved in 100% ethanol), followed by heating at 100°C for 45 min. One unit of enzyme activity is defined as the amount of enzyme that catalyzes the formation of 1 µmol of urea per min.

Amastigotes were purified from the lesions as described in [36] and arginase activity was measured as described above

### Determination of L-arginine concentration

Single cell suspensions from individual footpads and popliteal lymph nodes were prepared in 500 µl PBS on cell dissociation sieves. Suspensions were centrifuged 5 min at 1600 rpm and cell-free supernatants were centrifuged further at 14000 rpm for 10 min. The supernatants were frozen for further use.

Cell-free supernatants were prepared for amino acid analysis by deproteinisation using sulphosalicylic acid; cell-free supernatants were filtered using VectaSpin Micro 0.45 mm filters (Whatman International Ltd.). Free amino acids were separated by ion-exchange chromatography and quantified by postcolumn ninhydrin derivatisation using an amino acid analyser (AminoTac JLC-500/V). An internal standard, S-(2-aminoethyl)-L-cysteine hydrochloride and amino acid standard solutions (basics; acidics and neutrals) were supplied by Sigma.

To test the possibility that L-arginine could be metabolised by arginase during the preparation of the cell-free supernatants, we measured the level of urea produced in the presence or in the absence of exogenous L-arginine in footpad extracts from 4 week infected BALB/c mice. Following a two-hour incubation on ice, we measured 6.2±0.2 µg urea in the presence of exogenous L-arginine and 6.0±0.3 µg urea in the absence of L-arginine. These results show that L-arginine is not hydrolyzed during the preparation of the footpad extracts.

### Proliferation assay

*L. major* infected mice were treated with 1 mg bromodeoxyuridine (BrdU) (Sigma) i.p. once a day for the last four days before experiments were terminated. Draining lymph nodes from individual mice or footpads (pool of at least 2 footpads) were homogenised into single cell suspensions using cell dissociation sieves and the frequencies of CD4^+^BrdU^+^ T cells were determined directly *ex vivo*. Before surface labeling with anti-CD4 mAb (clone H129.19 or GK1.5, Pharmingen), cells were preincubated with 1 µg of rat anti-mouse monoclonal antibody CD32/CD16 (FcγII/III receptor, Pharmingen). Cells were washed, fixed and permeabilized using the method described in [37]. Detection of CD4^+^ BrdU^+^ cells was performed using a FACSCalibur (Becton Dickinson) and data were analyzed using Summit v4.3 software.

### Intracellular cytokine determination

Draining lymph nodes from individual mice or footpads (pool of at least 2 footpads) were prepared as described above. Cells (1×10^6^) were stimulated with 50 ng of phorbol 12-myristate 13-acetate (PMA; Sigma) and 500 ng of ionomycin (Calbiochem) or, as a control, in the presence of complete medium alone for 4 h, with 10 µg of brefeldin A (Sigma) added for the last 2 h. Before surface labeling with anti-CD4 mAb (clone H129.19 or GK1.5, Pharmingen), cells were preincubated with 1 µg of rat anti-mouse monoclonal antibody CD32/CD16 (FcγII/III receptor, Pharmingen). Cells were washed, fixed and permeabilized as described in [38] before the anti-cytokine antibodies or the isotype controls were added (anti-IL-4 mAb, clone BVD4-1D11; anti-IFN-γ mAb, clone XMG1.2; anti-IL-10 mAb, clone JES5-16E3; appropriately labeled rat immunoglobulin (Pharmingen)). Detection of intracellular cytokines was performed using a FACSCalibur (Becton Dickinson) and data were analyzed using Summit v4.3 software.

### Statistical analyses

Statistical differences were determined using a two-tailed Mann-Whitney test and differences were considered statistically significant at *P*<0.05.

## Results

### High levels of arginase activity are restricted to the site of pathology

**Figure 1:**
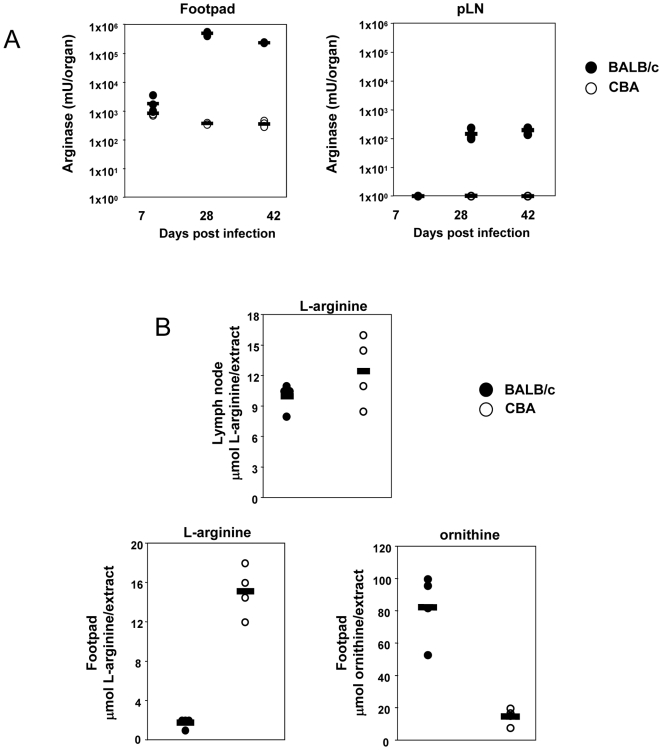
High arginase activity is localised at the site of pathology and results in reduced levels of L-arginine at the site of pathology. (A) Groups of BALB/c and CBA mice (n = 4) were infected with *L. major* parasites in one hind footpad and 1, 4 and 6 weeks post infection, individual footpads and popliteal lymph nodes were harvested and arginase activity was determined by enzymatic assay. Each symbol represents arginase activity for one individual organ, the horizontal line representing the average value. Data show the results of one representative experiment out of five independent experiments. (B) Groups of BALB/c and CBA mice (n = 4) were infected with *L. major* parasites in one hind footpad and 4 weeks post infection, individual footpads and popliteal lymph nodes were harvested and levels of extracellular L-arginine were determined using an amino acid analyser. Data show the results of one representative experiment out of two independent experiments.

In the experimental model of infection with *L. major*, parasite growth and pathology occur mainly at the site of infection, that is, in the footpads. Healer strains, such as CBA mice, develop small lesions at the site of infection, which heal spontaneously within a few weeks; by contrast, BALB/c mice, the prototypic nonhealer strain of mice, develop progressive nonhealing lesions. We have recently shown that arginase activity is significantly higher at the site of pathology in infected nonhealer BALB/c mice than in healer CBA mice [15]. Here, we extend these results and show that the expression of high arginase activity is restricted to the site of parasite growth and pathology, the infected footpads: in nonhealer mice, arginase activities were significantly higher in the lesions than in the lymph nodes draining the lesions (Figures 1A). In healer mice, low arginase activity was exclusively detectable in the footpads and was below the detection limit in the draining lymph nodes (Figure 1A). Arginase was below the detection limit in the spleen of both groups of mice and was not detectable in the contralateral non-infected footpad (data not illustrated). To account for the differences in organ sizes, we also determined arginase activity as mU/mg of protein and found similar tendencies as those in Figure 1 (data not illustrated).

We and others have shown that *Leishmania* parasites express their own arginase [39],[40]. We have already shown by enzymatic assay, Western blot and PCR that in the lesions of mice infected with *L. major*, as well as in infected bone marrow derived macrophages, arginase activity measured was mainly of host origin [15],[41]. To support these results further, we measured arginase in the lesions of BALB/c mice four weeks post infection, as well as in amastigotes purified from the lesions. Arginase activity was detectable in amastigotes (2.6±0.2 U/lesion), however, there was a 80.4-fold higher arginase expression in the lesions (209.2±12.8 U/lesion, data not shown), further confirming that the majority of arginase activity measured in the lesion is host arginase.

Next, we measured the levels of arginase activity in the lesions of BALB/c and CBA mice following infection with metacyclic *L. major* parasites [32]. As shown in Table 1, arginase activity is significantly higher at the site of pathology in infected BALB/c mice than in CBA mice.

**Table 1:** Infection with metacyclic *L. major* parasites results in significantly higher arginase activity in the lesions of BALB/c mice than CBA mice.

Arginase activity (mU/mg protein)	BALB/c	CBA
Day 7	1,521.8±89.2	452.1±23.9
Day 28	452,363.0±22,421.3	221.9±9.5
Day 42	426,978.6±19,125.3	198.6±5.6

Groups of BALB/c and CBA (n = 4) mice were infected with metacyclic *L. major* parasites in one hind footpad and 1, 4 and 6 weeks post infection, individual footpads were harvested and arginase activity was determined by enzymatic assay. Values given are the average arginase activity ±standard deviation. Data show the results of one representative experiment out of three independent experiments.

The results presented in Figure 1A show that high arginase activity is a hallmark of nonhealing disease and is localised at the site of pathology.

### High arginase activity at the local site of pathology induces depletion of L-arginine in the microenvironment

We and others have shown that induction of arginase in myeloid cells rapidly results in the depletion of L-arginine in the culture medium [15],[18],[23]. Moreover, higher arginase activity measured in PBMCs from patients suffering from tuberculosis [30] and asthma [42] coincides with lower levels of L-arginine in the plasma. In these studies, the levels of arginase activity and L-arginine were measured in the circulation, but not at the site of pathology. We wished to test the hypothesis that the high levels of arginase activity present in the lesions of nonhealing mice might result in local depletion of L-arginine from the extracellular milieu. We therefore measured the levels of L-arginine in the draining lymph nodes and lesions of *L. major*-infected healer and nonhealer mice. As shown in Figure 1B (upper panel), there was a small non-significant decrease in the levels of L-arginine in the extracts from draining lymph nodes of nonhealer mice 4 weeks post infection (12.5±3.4 vs 10.0±1.4 nmol/lymph nodes extract, *P*>0.05). In sharp contrast, the levels of L-arginine were found to be significantly lower in the lesion extracts of nonhealer mice than in healer mice (1.8±0.5 vs 15.1±2.5 nmol/footpad extract, *P*<0.05, Figure 1B, lower left panel). Consistent with this observed decrease in L-arginine concentrations in nonhealer lesions, we observed an increase in the levels of ornithine, one of the products of arginase-mediated L-arginine catabolism, in these lesions (Figure 1B, lower right panel). Similar results were obtained with lesions from healer and nonhealer mice 2 weeks post infection (data not illustrated). The levels of L-arginine found in the footpad extracts of non-infected healer and nonhealer mice were 22.0±2.1 and 18.5±4.4 nmol/footpad extract respectively (*P*>0.05, data not illustrated).

Since we cannot evaluate the volume of extra-cellular fluid in the lymph nodes and footpads, we also expressed the levels of L-arginine as a percentage of the combined concentrations (%L-arginine) of the ten essential amino acids (arginine, valine, histidine, isoleucine, leucine, lysine, methionine, phenylalanine, tryptophan and threonine,). Using this approach, we obtained similar results as those presented in Figure 1B. That is, a small reduction in %L-arginine was found in the draining lymph nodes of nonhealer mice as compared to healer mice (8.8±1.9% vs 13.9±0.9%, *P*<0.05, data not illustrated), whereas %L-arginine was considerably lower in the footpads of nonhealer mice as compared to healer mice (0.4±0.1% vs 8.6±1.6%, *P*<0.05, data not illustrated).

These results show that the high levels of arginase activity present at the site of pathology results in the depletion of L-arginine from the microenvironment.

### Depletion of L-arginine coincides with impaired proliferation of effector CD4^+^ T cells at the site of pathology

**Figure 2:**
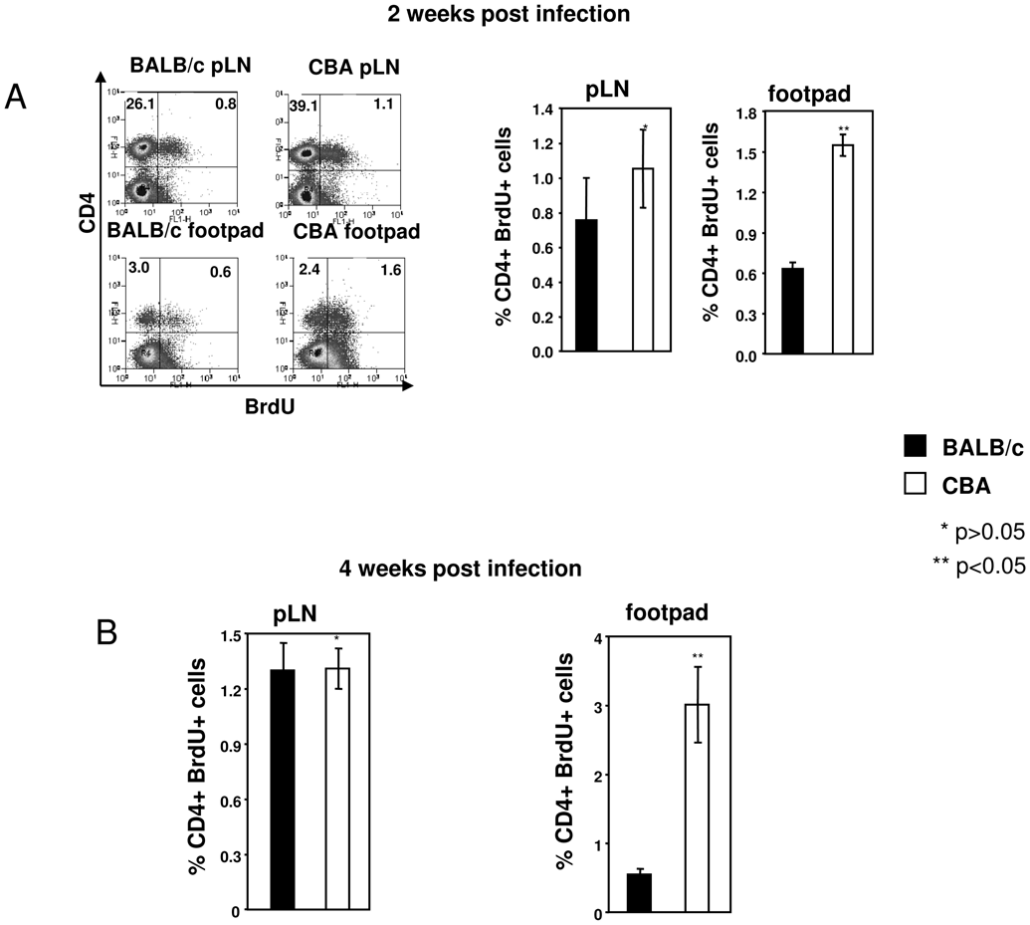
Impaired proliferation of antigen-specific CD4^+^ T cells. Groups of BALB/c and CBA mice (n = 8) were infected with *L. major* parasites for two (A) or four (B) weeks and were injected with 1 mg BrdU daily for 4 days. Individual popliteal lymph nodes and footpads (pool from at least two footpads) were harvested and the % of proliferating CD4^+^ T cells was determined by flow cytometry. (2A) left panels: dot plot profiles of CD4^+^BrdU^+^ T cells 2 weeks post infection; right panels: % of CD4^+^ BrdU^+^ T cells 2 weeks post infection. (2B) % of CD4^+^ BrdU^+^ T cells 4 weeks post infection. Error bars represent standard deviations. Isotype control: 0.27%. Data show the results of one representative experiment out of three independent experiments.

We then assessed the impact of the reduced levels of L-arginine in the lesions on local T cell responses: we hypothesized that, in the lesions of nonhealer mice, high arginase activity and the consequent low level of L-arginine (Figures 1A and B) result in impairment of T cell responses, whereas the T cell responses are unaffected in the draining lymph nodes where arginase activity was low or undetectable (Figures 1A). To test this hypothesis, we first measured the proliferation of *L. major*-specific CD4^+^ T cells *in vivo* by quantifying the frequency of CD4^+^ T cells that had incorporated BrdU *in vivo*, in cells isolated from the draining lymph nodes and the lesions, without any further restimulation. We found that, two weeks post infection, there was a small and non-significant increase in the frequency of proliferating CD4^+^ T cells in the draining lymph nodes of healer mice compared with nonhealer mice (1.05±0.2% vs 0.76±0.2%, *P*>0.05, Figures 2A). However, at the site of pathology, the frequency of CD4^+^BrdU^+^ T cells was significantly greater in healer mice than in nonhealer mice (1.55±0.1% vs 0.63±0.2%, *P*<0.05) (Figures 2A). Similarly, the frequency of proliferating CD4^+^ T cells was significantly greater in the lesions of healer mice than in nonhealer mice 4 weeks post infection (Figure 2B, 3.0±0.6% vs 0.6±0.1%, *P*<0.05); in contrast the frequency of proliferating CD4^+^ T cells in the lymph nodes did not differ between the two strains 2 and 4 weeks post infection (Figure 2A and B). These results show that when measured directly *ex vivo*, the frequency of proliferating CD4^+^ T cells in the draining lymph nodes *in vivo* was similar in healer and nonhealer strains of mice. In sharp contrast, the *in vivo* frequency of proliferating CD4^+^ T cells was significantly lower in the lesions of nonhealer mice than in those of healer mice.

It is unlikely that the impaired proliferation observed in the lesions of nonhealer mice is due to different abilities of lymphocytes to migrate to the lesions. Indeed, cells migrate even more efficiently in nonhealer BALB/c mice as shown by a significantly increased cell infiltration in their lesions compared to healer CBA mice (132±9×10^4^ vs 80±5×10^4^ cells/lesions, data not illustated). This represents 4.8×10^4^ CD4^+^ T cells in the lesions of BALB/c mice as compared to 3.2×10^4^ in CBA mice. These results show that there is a higher frequency of CD4^+^ T cells migrating to the lesions in nonhealer mice and therefore show that the migration of CD4^+^ T cells to the lesions is not impaired in BALB/c mice.

Of note, whereas there are more CD4^+^BrdU^+^ T cells in the draining lymph node as compared to the footpads (Figure 2A), the frequency of proliferating CD4^+^ T cells is clearly higher in the footpad: indeed, 16.7% of CD4^+^ T cells (0.6% of 3.6%) are proliferating in the lesions of BALB/c mice as compared to 2.9% (0.8% of 26.9%) in the draining lymph nodes; in CBA mice, 40% of CD4^+^ T cells in the lesions are BrdU^+^ (1.6% of 4%) vs 2.7% in the draining lymph nodes (1.1% of 40.2%).

The results presented in Figures 1 and 2 show that high arginase activity at the local site of infection and the subsequent low L-arginine levels in nonhealer mice are accompanied by reduced proliferation of CD4^+^ T cells in the lesion.

### Depletion of L-arginine is associated with impaired cytokine production by effector CD4^+^ T cells at the site of pathology

**Figure 3:**
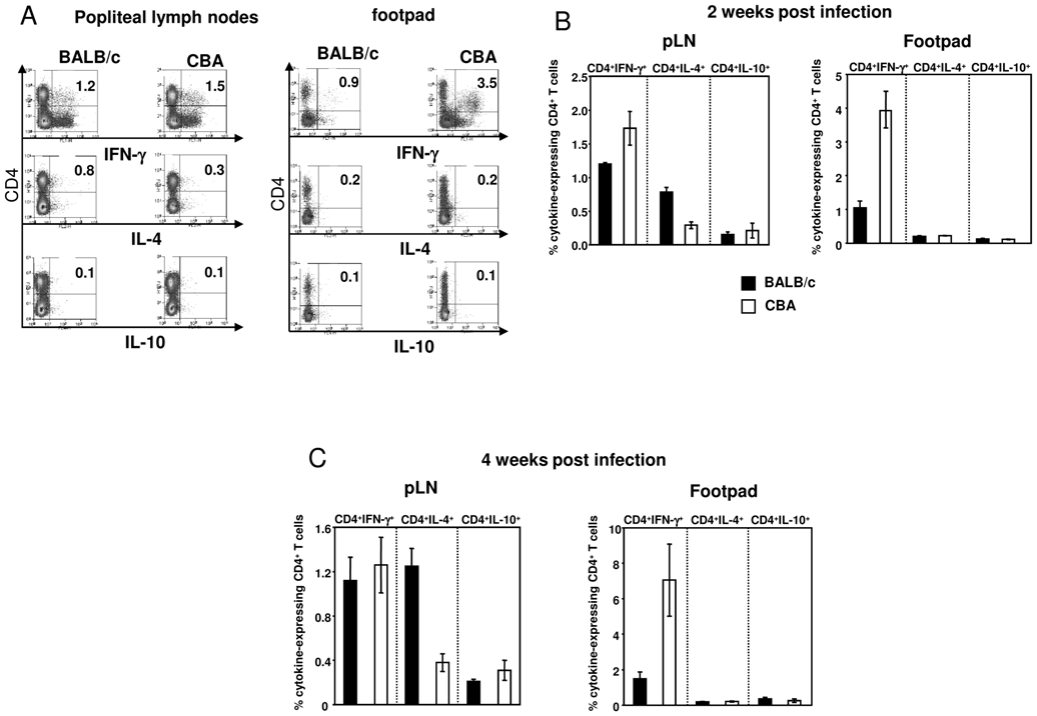
Impaired capacity of antigen-specific CD4^+^ T cells to express cytokines. Groups of BALB/c and CBA mice (n = 8) were infected with *L. major* parasites for two (A and B) or four (C) weeks. Individual popliteal lymph nodes and footpads (pool from at least two footpads) were harvested and the % of cytokine-expressing CD4^+^ T cells was determined by flow cytometry. (3A) dot plot profiles of cytokine-expressing CD4^+^ T cells isolated from the popliteal lymph nodes and the footpads two weeks post infection; (3B) % of cytokine-expressing CD4^+^ T cells two weeks post infection. (3C) % of cytokine-expressing CD4^+^ T cells four weeks post infection. Error bars represent standard deviations. Isotype control for IFN-γ: 0.23±0.05%, IL-4: 0.31±0.08% and IL-10: 0.26±0.06%. All cytokines were below the detection limit when the cells were stimulated in the absence of PMA/ionomycin stimulation. The detection limits were defined as % isotype control +3 standard deviations: IFN-γ = 0.38%, IL-4 = 0.55% and IL-10 = 0.44%. Data show the results of one representative experiment out of three independent experiments.

Cytokines play an important role in both the healing and the nonhealing forms of experimental leishmaniasis. Therefore, we investigated the frequency of *L. major*-specific IFN-γ^+^, IL-4^+^ and IL-10^+^ CD4^+^ T cells directly *ex vivo*, both in the draining lymph nodes and in the lesions of infected nonhealer (BALB/c) and healer (CBA) mice. As shown in Figures 3A and B, CD4^+^IFN-γ^+^ T cells were detectable in the lymph nodes draining the lesions in both strains of mice, with a small increase in the frequency of CD4^+^IFN-γ^+^ T cells in healer mice (1.7±0.3% vs 1.2±0.1%, *P*<0.05). CD4^+^IL-4^+^ T cells were detectable in the draining lymph nodes of nonhealer mice, but not in healer mice, and IL-10 was below the detection limit in both groups. Although no intracellular IL-4 or IL-10 was detectable in the lesions of either BALB/c or CBA mice, CD4^+^ IFN-γ^+^ T cells were detectable in the lesions of *L. major*-infected mice from both nonhealer and healer strains (Figures 3A and B). However, there was a markedly higher frequency of CD4^+^IFN-γ^+^ T cells in the lesions of healer than nonhealer mice (3.9±0.5% vs 1.1±0.2%, *P*<0.05). Similar results were obtained 4 weeks post infection (Figure 3C).

These results show that antigen-specific CD4^+^ T cells at the site of pathology in nonhealing lesions have an impaired capacity to express IFN-γ, whereas this response is only mildly affected in the draining lymph nodes.

We conclude that high arginase activity at the local site of infection and the consequent low L-arginine levels in nonhealer mice are associated with an impaired capacity of CD4^+^ T cells to produce IFN-γ.

### Cure of nonhealing leishmaniasis correlates with downregulation of arginase activity and restoration of CD4^+^ T cell responses

As a further test of the hypothesis that high arginase activity correlates with local immunosuppression and to test the prediction that downregulation of arginase correlates with restoration of effector responses, we investigated T cell functions in BALB/c mice that were chemotherapeutically treated to control the infection. This model would also exclude any contribution of genetic factors that might cause the different outcomes of disease in different strains of mice.

**Figure 4:**
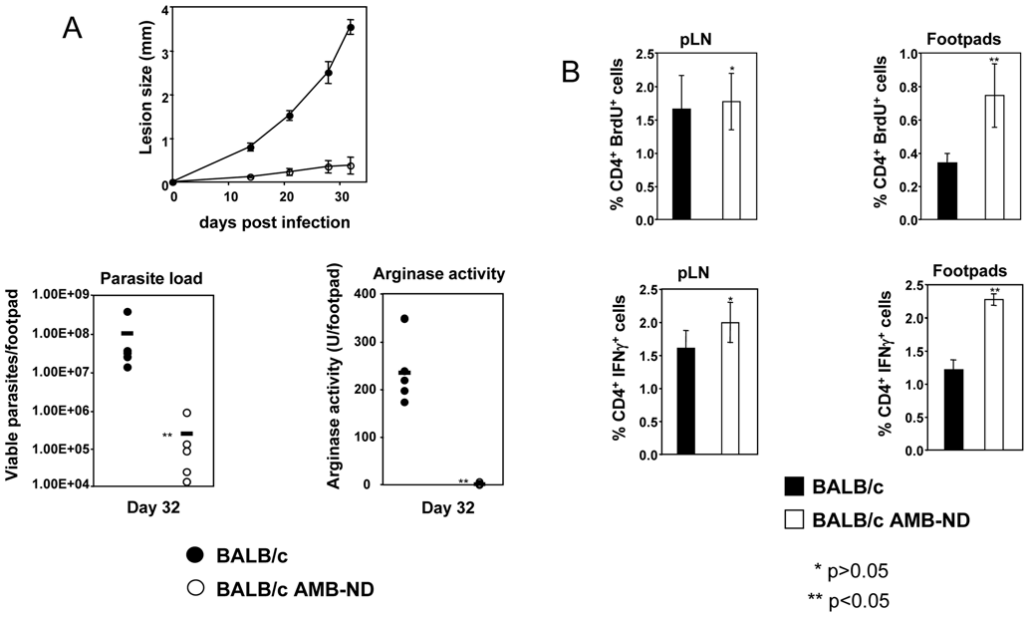
Control of disease in BALB/c mice correlates with downregulation of arginase and restoration of immune responses. Two groups of BALB/c mice (n = 5) were infected with *L. major* parasites in one hind footpad and one group was injected i.p. with 5 mg/kg of AMB-ND on day 1, 2, 7, 14, 21 and 28. (A) The lesion development was monitored at weekly intervals and five weeks post infection, number of viable parasites and the arginase activity was measured in individual footpads. (B) % of CD4^+^ BrdU^+^ T and % of IFN-γ-expressing CD4^+^ T cells in individual popliteal lymph nodes and lesions (pool of at least 2 footpads), error bars represent standard deviations. Data show the results of one representative experiment out of two independent experiments.

In this study, we used a novel lipid formulation of the macrolide polyene antibiotic, amphotericin B (AMB) [34]. Recent studies have shown that nanoscale, discoidal particles of reconstituted high-density lipoprotein, enriched with AMB (termed nanodisks (ND)) induce clearance of *L. major* infection in BALB/c mice [35]. As shown here in Figure 4A, AMB-ND-treated mice developed only minor lesions 5 weeks post infection, whereas untreated *L. major*-infected BALB/c mice developed ulcerated lesions that contained >400-fold more parasites (*P*<0.05). Significantly lower arginase activity was observed in the lesions of AMB-ND-treated BALB/c mice (Figure 4A, *P*<0.05), confirming further that low arginase expression correlates with control of parasite replication [15].

To assess whether low arginase activity in the lesions of healed BALB/c mice was associated with restored immune effector functions, we isolated both lesions and draining lymph nodes from AMB-ND treated and control infected BALB/c mice and measured the capacity of CD4^+^ T cells to proliferate and express IFN-γ directly *ex vivo*. We found that CD4^+^ T cells from draining lymph nodes from both groups of mice had a similar capacity to proliferate and express IFN-γ (Figure 4B). In contrast, there was a significant increase in the capacity of CD4^+^ T cells from the lesions of BALB/c mice treated with AMB-ND both to proliferate and express IFN-γ (Figure 4B).

The results presented in Figure 4 show that healing is associated with a significant reduction in arginase activity at the site of pathology and with restoration of efficient CD4^+^ T cell effector functions *in vivo*.

### Interfering with L-arginine metabolism restores CD4^+^ T cell responses at the site of pathology

**Figure 5:**
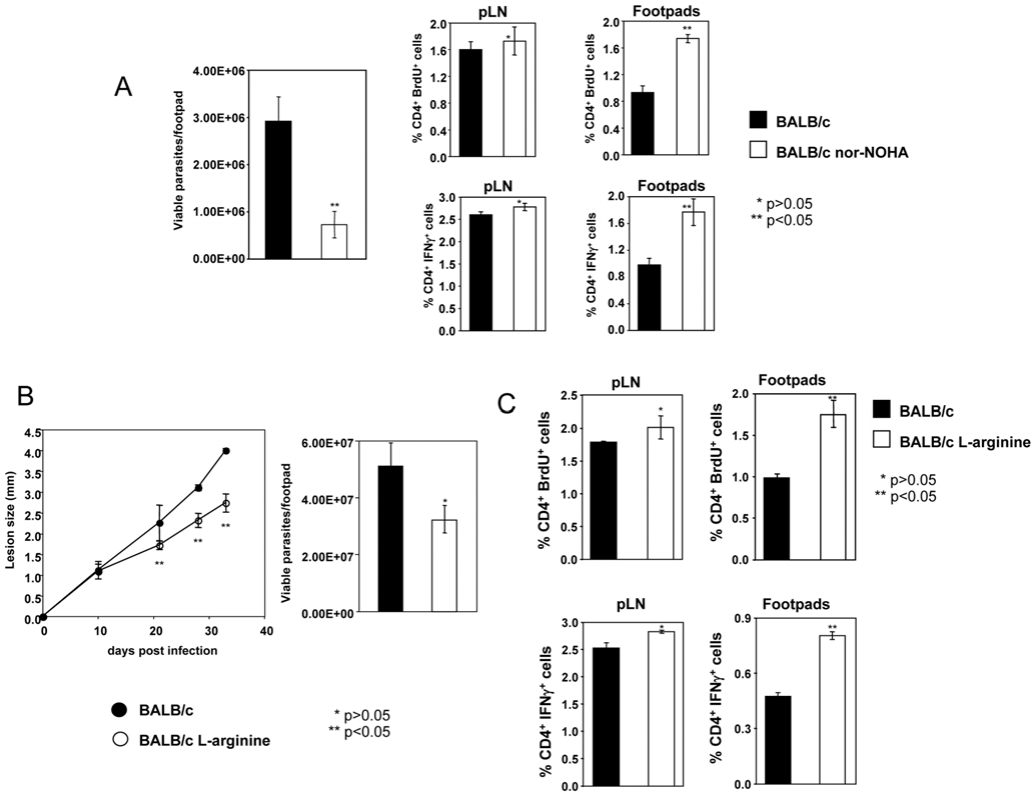
Interfering with arginase-induced L-arginine metabolism restores CD4^+^ T cell responses in nonhealer BALB/c mice. (A) Two groups of BALB/c mice (n = 8) were infected with *L. major* parasites and one group was injected daily i.p. with a competitive inhibitor of arginase (nor-NOHA, 1 mg/mouse) starting from the day of infection. Two weeks later, the number of viable parasites was measured in individual footpads (left panel) and the % of CD4^+^ BrdU^+^ T cells and IFN-γ-expressing CD4^+^T cells was determined by flow cytometry in popliteal lymph nodes and lesions (pool of at least 2 footpads). Error bars represent standard deviations. Data show the results of one representative experiment out of two independent experiments. (B) Two groups of BALB/c mice (n = 8) were infected with *L. major* parasites in one hind footpad and 2 weeks post infection, one group was injected i.p. with L-arginine (10 mg/mouse) 3 times a week, for 3 weeks. The lesion development was monitored at weekly intervals (left panel) and 33 days post infection, number of viable parasites and the arginase activity was measured in individual footpads (right panel). (C) % of CD4^+^ BrdU^+^ T cells and IFN-γ-expressing CD4^+^T cells in popliteal lymph nodes and lesions (pool of at least 2 footpads), error bars represent standard deviations. Data show the results of one representative experiment out of four independent experiments.

To demonstrate further that arginase-induced L-arginine depletion results in impaired T cell responses at the site of pathology, we treated *L. major*-infected nonhealer BALB/c mice with a competitive inhibitor of arginase, *N* ^ω^-hydroxy-nor-L-arginine (nor-NOHA) [15]. Two weeks of treatment with nor-NOHA resulted in non-significant differences in lesion size (1.4±0.2 vs. 1.2±0.1 mm, P>0.05, data not shown) and arginase activity (16.8±2.9 vs 16.0±4.9 mU/footpad, P>0.05, data not illustrated) and significantly reduced parasite load at the site of pathology (Figure 5A, P<0.05). We can exclude the possibility that nor-NOHA is responsible for the lower parasite load as we have show previously that it does not affect parasite growth [15]. Importantly, the proliferation and production of IFN-γ by CD4^+^ T cells was significantly higher in lesions of mice that were treated with the competitive inhibitor of arginase (P<0.05, Figure 5A). No differences were observed in the draining lymph nodes (Figure 5A). These results show that *in vivo* inhibition of arginase activity restores CD4^+^ T cell responses at the site of pathology.

Finally, to demonstrate unequivocally that L-arginine depletion at the site of pathology is responsible for T cell hyporesponsiveness, BALB/c mice were treated with L-arginine starting from day 10 post infection. As shown in Figure 5B, five weeks post infection, BALB/c mice treated with L-arginine develop significantly smaller lesions and harbour less parasites in their lesions as compared to the untreated group. Arginase activity was lower in the treated group than in the untreated group (165±41 vs 194±26 U/footpad respectively, P>0.05, data not illustrated). To determine whether treatment with L-arginine supplementation restores T cell responses, we measured proliferation and IFN-γ production by CD4^+^ T cells in the draining lymph nodes and in the lesions. We found that there was a significant increase in the capacity of CD4^+^ T cells from the lesions of BALB/c mice treated with L-arginine both to proliferate and express IFN-γ as compared to the untreated group (Figure 5C). CD4^+^ T cells from draining lymph nodes from the control group of mice and that treated with L-arginine had a similar capacity to proliferate and express IFN-γ (Figures 5C).

These results show that treatment of nonhealer BALB/c mice with L-arginine restores CD4^+^ T cell effector functions at the site of pathology.

## Discussion

One of the main immunological characteristics of the active form of visceral leishmaniasis is the inability of peripheral blood mononuclear cells to mount an efficient immune response, as shown by their impaired capacity to proliferate or produce IFN-γ in response to *Leishmania* antigen *in vitro* [9],[10],[11],[43]. PBMCs from patients with recurrent cutaneous infections also produce lower levels of IFN-γ than those from patients with active lesions or patients who had recovered [44]. PBMCs isolated from patients with diffuse cutaneous leishmaniasis are hyporesponsive to antigenic restimulation [12],[45],[46]. In sharp contrast, PBMCs from patients affected by the self-healing form of cutaneous leishmaniasis proliferate and produce IFN-γ when stimulated with *Leishmania* antigen [44],[47],[48]. The general picture emerging from the studies described above is that immunosuppression is a hallmark of nonhealing forms of cutaneous and visceral leishmaniasis. However, the molecular mechanisms responsible for immunosuppression in the nonhealing form of leishmaniasis are not understood.

The ability of T cells to proliferate and produce cytokines in response to antigenic challenge has been shown by restimulating PBMCs with *Leishmania* antigens *in vitro*. Similarly, in the mouse models of both cutaneous and visceral leishmaniasis, immune responses have mostly been evaluated by restimulating lymph node or spleen cells with *Leishmania* antigen *in vitro*. By assaying the effector functions of antigen-specific CD4^+^ T cells isolated from the site of pathology, without further restimulation, we show here that CD4^+^ T cells isolated from the lesions of nonhealer BALB/c mice proliferate at a significantly lower rate than those from self-healing CBA or BALB/c mice cured chemotherapeutically. CD4^+^ T cells isolated from diseased mice also have an impaired ability to express IFN-γ. These results substantiate the observation made in the different forms of nonhealing leishmaniasis in humans, that severe disease is associated with inefficient immune responses. Our results suggest a mechanism for the observed impaired immune responses in leishmaniasis: in the lesions, local depletion of L-arginine by arginase impairs the ability of T cells to mount an effective response.

L-arginine depletion by arginase-expressing myeloid cells has been shown previously *in vitro* [15],[23],[49],[50]; and a correlation between high arginase activity and reduced levels of L-arginine has been shown in the plasma of patients suffering from active tuberculosis [30]. Arginase-mediated L-arginine deprivation has been shown to cause T cell hyporesponsiveness in both pathological and physiological responses [22],[24],[25],[51]. Further, diseases such as chronic inflammation [23], asthma [26], psoriasis [27] and certain infectious diseases such as schistosomiasis [28], trypanosomiasis [29], leishmaniasis [15] and tuberculosis [30] have been associated with increased arginase activity. However, the effects of high arginase activity on the bioavailability of L-arginine were not studied at the local site of pathology. Here, we show that L-arginine is depleted from the microenvironment at the site of pathology and that this low level of L-arginine results in local immunosuppression *in vivo* in nonhealing leishmaniasis. T cell responses were most strongly impaired at the site of pathology - in the footpads - whereas only minor downregulation of the immune response was detected in the draining lymph nodes. In the latter, arginase levels were low or below the detection limit, and levels of L-arginine were unaltered. Both the proliferation and the capacity of CD4^+^ T cells to express IFN-γ were significantly lower in the lesions of nonhealer mice than in those of healer mice. To confirm further that this T cell hyporesponsiveness is induced by arginase-induced L-arginine depletion, we interfered with L-arginine metabolism *in vivo* by inhibiting arginase or by supplementing the mice with L-arginine. In both instances, lesion development and parasite load were reduced and CD4^+^ T cell responses were restored at the site of pathology. Therefore, we conclude that nonhealing lesions and uncontrolled parasite growth are caused by arginase-mediated L-arginine depletion, which in turn impairs proliferation and prevents CD4^+^ T cells from expressing IFN-γ and inducing efficient killing of intracellular parasites in macrophages.

It is well established that *Leishmania* parasites express arginase [15],[39],[40]. Although our previous work as well as the data presented here show that the majority of arginase detected in cutaneous lesions is host arginase [15],[41], it is possible that parasite arginase also modulates the host response [52].

The results presented here substantiate our previous study, which showed that competitive inhibition of arginase enables BALB/c mice to control the lesion development, pathology and parasite load more efficiently [15]. The mice treated with nor-NOHA displayed smaller lesion sizes and arginase activities were lower at the site of pathology, however, differences were not statistically significant between the treated and untreated groups; this may be due to the timing as these experiments were performed for 2 weeks. Further, Holscher et al. also showed that delayed disease onset in macrophage/neutrophil-specific IL-4R-deficient mice was accompanied by reduced levels of arginase [53].

Here we show that low levels of IL-4 were detectable in the draining lymph nodes of *L. major*-infected BALB/c mice directly *ex vivo* and but was below detection limit in the lesions. It is possible that flow cytometry is not sensitive enough to detect CD4^+^ T cells that express low levels of IL-4 and/or in addition, there was a only a small number of cells in the lesions. Th1 and Th2 responses associated with healing and nonhealing, respectively [1],[2],[54], have been largely defined by measuring cytokine production following *in vitro* restimulation of lymphoid cells with *Leishmania* parasites. We have recently shown that T helper cell responses are significantly less polarized when determined *ex vivo* as compared to those measured after restimulation *in vitro* [55]. Notably, the difference in IL-4 production by CD4^+^ T cells between nonhealer and healer mice was 2.0-fold when determined *ex vivo* and 21.1-fold when measured following *in vitro* restimulation [55]. Therefore, whereas lymphoid cells from nonhealer mice mount a strong IL-4 response after *in vitro* restimulation, the production of IL-4 by CD4^+^ T cells directly *ex vivo* is low.

Arginase is an essential enzyme and indeed, arginase knockout mice die by two weeks of age from hyperammonemia. Therefore, even though our previous study [15] showed promising results when targeting arginase activity systemically with a competitive inhibitor, it is unlikely that inhibition of arginase activity could be used as an intervention strategy. However, dissecting the mechanisms responsible for the induction of local arginase is likely to provide new targets for the control of this pathway. Towards this end, the dramatic effects of AMB-ND therapy on lesion size, parasite burden and arginase activity in the nonhealer strains of mice not only provides a useful experimental system, but also a potentially viable therapy for treatment of *Leishmania* infection. The mechanisms resulting in the control of arginase activities at the site of pathology following treatment with AMB-ND are not fully understood. Here we show that efficient control of parasite replication in the lesions was associated with control of arginase activity. We have previously shown that infection of macrophages with *L. major* parasites strongly synergizes with IL-4 to increase arginase activity [56] and we have shown that the arginase measured was mainly host arginase [15]. Importantly, infection of macrophages by *L. majo*r alone results in the induction of arginase [56],[15]. Therefore, it is tempting to speculate that in addition to factors such as cytokines, the parasites themselves act to induce and maintain arginase expression in macrophages. This suggests that high expression of arginase activity is dependent on the viability of the parasites and that to efficiently control arginase, parasites replication has to be restricted. Treatment with L-arginine might also be a promising therapeutic avenue. Indeed, our results show that treatment with L-arginine not only restores efficient T cell responses, but also improves pathology and decreases parasite load. It has already been shown that treatment with L-arginine has a potential role in immunomodulation; benefits include enhanced T cell functions, increase in macrophage cytotoxicity and phagocytosis and accelerated wound healing potential [57],[58],[59].

The findings presented here provide a novel mechanism that explains the incapacity of CD4^+^ T cells to mount an efficient response in the nonhealing form of leishmaniasis. Targeting the metabolism of L-arginine is likely to be an important therapeutic and prophylactic strategy to treat not only leishmaniasis, but also other diseases associated with high arginase activity such as cancer, psoriasis and allergic asthma.
